# Prospective Whole-Genome Sequencing Enhances National Surveillance of Listeria monocytogenes

**DOI:** 10.1128/JCM.02344-15

**Published:** 2016-01-28

**Authors:** Jason C. Kwong, Karolina Mercoulia, Takehiro Tomita, Marion Easton, Hua Y. Li, Dieter M. Bulach, Timothy P. Stinear, Torsten Seemann, Benjamin P. Howden

**Affiliations:** a Doherty Applied Microbial Genomics, Department of Microbiology and Immunology, University of Melbourne at the Doherty Institute for Infection & Immunity, Melbourne, Victoria, Australia; b Microbiological Diagnostic Unit Public Health Laboratory, Department of Microbiology and Immunology, University of Melbourne at the Doherty Institute for Infection & Immunity, Melbourne, Victoria, Australia; c Infectious Diseases Department, Austin Health, Heidelberg, Victoria, Australia; d Victorian Life Sciences Computation Initiative, University of Melbourne, Victoria, Australia

## Abstract

Whole-genome sequencing (WGS) has emerged as a powerful tool for comparing bacterial isolates in outbreak detection and investigation. Here we demonstrate that WGS performed prospectively for national epidemiologic surveillance of Listeria monocytogenes has the capacity to be superior to our current approaches using pulsed-field gel electrophoresis (PFGE), multilocus sequence typing (MLST), multilocus variable-number tandem-repeat analysis (MLVA), binary typing, and serotyping. Initially 423 L. monocytogenes isolates underwent WGS, and comparisons uncovered a diverse genetic population structure derived from three distinct lineages. MLST, binary typing, and serotyping results inferred *in silico* from the WGS data were highly concordant (>99%) with laboratory typing performed in parallel. However, WGS was able to identify distinct nested clusters within groups of isolates that were otherwise indistinguishable using our current typing methods. Routine WGS was then used for prospective epidemiologic surveillance on a further 97 L. monocytogenes isolates over a 12-month period, which provided a greater level of discrimination than that of conventional typing for inferring linkage to point source outbreaks. A risk-based alert system based on WGS similarity was used to inform epidemiologists required to act on the data. Our experience shows that WGS can be adopted for prospective L. monocytogenes surveillance and investigated for other pathogens relevant to public health.

## INTRODUCTION

Listeria monocytogenes is a predominantly food-borne pathogen capable of causing a range of clinical illnesses, including invasive disease such as bacteremia and meningoencephalitis in humans, and is commonly monitored by public health facilities for the emergence of outbreaks (1, 2). A number of serotypes of L. monocytogenes can be isolated from environmental and food sources, but most outbreaks of human disease are due to serotypes 1/2a, 1/2b, and 4b (3).

Whole-genome sequencing (WGS) has emerged as a powerful technology for the comparison of isolates in outbreak analysis. Although proof-of-concept studies have been published to demonstrate the applications of WGS in clinical and public health microbiology, they have largely been conducted retrospectively or in response to an emerging outbreak. Despite calls for real-time genomics-based pathogen surveillance (4), only a few studies have reported the use of WGS in prospective surveillance and in the typing of bacteria to date (5, 6).

The Microbiological Diagnostic Unit Public Health Laboratory is the Australian Listeria reference laboratory and routinely performs molecular typing of human and nonhuman isolates of L. monocytogenes referred from local and interstate laboratories. We evaluated the use of routine prospective WGS compared with the use of conventional typing methods, including pulsed-field gel electrophoresis (PFGE), multilocus sequence typing (MLST), multilocus variable-number tandem-repeat analysis (MLVA), binary typing, and PCR serotyping, for the national epidemiologic surveillance of L. monocytogenes.

## MATERIALS AND METHODS

### Conventional typing. (i) Multilocus sequence typing.

MLST was performed via the PCR amplification of seven housekeeping genes (*acbZ*, *bglA*, *cat*, *dapE*, *dat*, *ldh*, and *lhkA*) per Institut Pasteur protocols (http://www.pasteur.fr/recherche/genopole/PF8/mlst/Lmono.html) until 2013. PCR products were purified using FastAP chemistry (Thermo Fisher Scientific, Waltham, MA, USA) and products sequenced on the ABI 3130x1 genetic analyzer using BigDye v3.1 chemistry (Applied Biosystems, Waltham, MA, USA). MLST profiles were analyzed in BioNumerics v7.5 (Applied Maths, Sint-Martens-Latem, Belgium) using the MLST plugin. Since 2012, *in silico* MLST has been inferred from WGS reads using SRST/SRST2 (v0.1.0 to v0.1.5) (7).

### (ii) Multilocus variable-number tandem-repeat analysis.

MLVA was performed using an Australian MLVA scheme developed in 2011 based on an optimized panel of nine previously reported variable-number tandem-repeat regions (8). The Lindstedt scheme was performed in parallel for international comparison (9). Fragments were amplified in two multiplex reactions using fluorescently labeled forward primers (Applied Biosystems). PCR products were diluted 1:10 with 1 μl of this dilution mixed with 12 μl formamide and 1 μl Geneflo 625 (ROX-labeled) internal size standard. Products were denatured at 95°C for 5 min, and capillary electrophoresis was performed on a 3130xL genetic analyzer (Applied Biosystems). Products were analyzed in BioNumerics v7.5 (Applied Maths) using the MLVA plugin.

### (iii) Binary typing and serotyping.

Established methods, using an eight-loci multiplex PCR panel for binary typing and a seven-loci multiplex PCR panel for serotyping, were used as previously described by others (10, 11). Binary typing targets were amplified in four multiplex reactions and were separated on a 3% agarose gel. PCR serotyping fragments were amplified in a single multiplex reaction using the primers in Table S2 in the supplemental material and were separated on a 2% agarose gel. The two gels were stained with GelRed and visualized on the Bio-Rad Gel Doc XR imaging system (Bio-Rad Laboratories, Hercules, CA, USA).

### (iv) Pulsed-field gel electrophoresis.

PFGE was performed by the published method of Carriere et al. with minor modifications (12). DNA was digested with restriction enzymes ApaI, SmaI, and NotI separately before running the gel for 22 h with a 5–25 s switch time. PFGE pattern numbers were assigned systematically as new patterns emerged.

### Whole-genome sequencing.

Cultures of L. monocytogenes isolated from food, environmental or clinical specimens, or from frozen glycerol storage were purified by two successive single colony selections after streaking onto horse blood agar incubated for 18 to 24 h at 37°C. Pure colonies were suspended in 200 ml of 20 mM Tris-HCl (pH 8.0), 2 mM EDTA, 1.2% Triton X-100, and 20 mg/ml lysozyme and were incubated for 30 min at 37°C. DNA was then extracted using the QIAamp DNA minikit (Qiagen, Hilden, Germany) or the JANUS Chemagic Workstation with the Chemagic Viral DNA/RNA kit (CMG-1033; PerkinElmer, Waltham, MA, USA).

Whole-genome sequencing was performed on the Illumina MiSeq or NextSeq platforms using Nextera XT libraries and protocols (Illumina, San Diego, CA, USA) with a minimum Phred quality score of 30. A sequencing depth of >30× was initially targeted but was later adjusted to >50× in 2014.

### Bioinformatic analyses of whole-genome sequencing data.

Sequencing reads were trimmed to clip Illumina Nextera adapters and low-quality sequences (Phred scores of <10) and were initially mapped to the reference genome F2365 (BioSample accession no. SAMN02603980) using Snippy v2.5/BWA-MEM v0.7.12 (https://github.com/tseemann/snippy). Variants were called using Snippy v2.5/Freebayes v0.9.21-7 (https://github.com/tseemann/snippy), requiring a minimum base quality of 20, a minimum read coverage of 10×, and a 90% read concordance at a locus for a variant to be reported. The pan-genome included all loci with a minimum read depth of 10× and comprised the core genome—the conserved nucleotide positions present in all isolates included in the analysis—and the accessory genome, which included all other nonconserved nucleotide positions. An alignment of core genome single nucleotide polymorphisms (SNPs) was produced in Snippy v2.5 to infer a phylogeny. The initial phylogenetic tree was constructed in SplitsTree4 (13) using neighbor-joining methods and was compared to a maximum-likelihood tree approximated using FastTree v2.1.8 (14). FastTree was run using the generalized time-reversible (GTR) model of nucleotide evolution and incorporated the CAT model to account for evolutionary rate heterogeneity across sites. Bootstrapping was performed by feeding 1,000 resampled alignments generated in SEQBOOT v3.69 (http://evolution.genetics.washington.edu/phylip/doc/seqboot.html) into FastTree using the -n option. Figtree v1.4.2 was used to view the resulting phylogenetic trees. A hierarchical Bayesian analysis of population structure (hierBAPS) (15) clustering model was also used to support phylogenetic groupings by using iterative clustering to a depth of 10 levels and a prespecified maximum of 20 clusters. For further phylogenetic comparison of isolates, sequence reads were remapped to the most closely related reference genome where possible, usually from the same MLST/clonal complex (CC). The relatedness of strains based on core genome SNP phylogenies was compared to traditional typing methods.

For *in silico* genome analysis, after determining optimal assembly *k*-mer size (16), reads were assembled *de novo* into scaffolds using Velvet v1.2.10 (17). Poor assemblies (≥400 contigs; *N*_50_, <15,000; comprising approximately the poorest 10% of the assemblies) were reassembled using SPAdes v3.5 (18) or A5-miseq v20141120 (19, 20) if the SPAdes assembly still had poor metrics or if typing information was incomplete due to assembly breaks, e.g., fragmented MLST alleles. Genome auto-annotation was performed with Prokka v1.11 using the F2365 (BioSample accession no. SAMN02603980) and EGD-e (BioSample accession no. SAMEA3138329) reference annotations (21). *Geneious* v7.1.5 (Biomatters Limited, Auckland, New Zealand) was used for genome and alignment visualization.

### Reference genomes.

Selected high-quality reference genomes were retrieved from the National Center for Biotechnology Information's (NCBI) GenBank repository and included in analyses for comparison (see Table S3 in the supplemental material).

### *In silico* typing.

Predictions of conventional typing results were made *in silico* from *de novo* assembled genomes. A bioinformatic tool for *in silico* serogrouping was developed based on EMBOSS Primersearch v6.6.0.0 (http://emboss.sourceforge.net/apps/) and isPCR v33 (http://hgwdev.cse.ucsc.edu/∼kent/src/) using the primers in Table S2 in the supplemental material. This tool, LisSero v0.1, is publicly available (https://github.com/MDU-PHL). *In silico* typing results were compared to the results from conventional typing where these were known. Where these results were discordant, the sequences were reassembled and further investigated using BLAST (http://blast.ncbi.nlm.nih.gov/Blast.cgi) to identify the presence of predicted primer amplicons and were also examined for read mapping coverage across the serotyping primer target and amplicon regions against selected reference genomes—EGD-e, serotype 1/2a (BioSample accession no. SAMEA3138329); Finland 1998, serotype 3a (BioSample accession no. SAMN00012880); R2-502, serotype 1/2b (BioSample accession no. SAMN02203126); SLCC2540, serotype 3b (BioSample accession no. SAMEA2272785); FSL R2 561, serotype 1/2c (BioSample accession no. SAMN00013319); SLCC2479, serotype 3c (BioSample accession no. SAMEA2272506); HCC23, serotype 4a (BioSample accession no. SAMN02603154); F2365, serotype 4b (BioSample accession no. SAMN02603980); SLCC2376, serotype 4c (BioSample accession no. SAMEA2272177); ATCC 19117, serotype 4d (BioSample accession no. SAMEA2271997); SLCC2378, serotype 4e (BioSample accession no. SAMEA2272689) (see Table S3 in the supplemental material).

*In silico* MLST was performed using a custom BLAST-based tool (https://github.com/tseemann/mlst) on *de novo* genome assemblies using the Listeria MLST allele database curated at the Institut Pasteur (http://www.pasteur.fr/recherche/genopole/PF8/mlst/Lmono.html). Clonal complexes were determined by grouping multilocus genotypes that shared 6 or more identical alleles of the 7 loci (*abcZ*, *bglA*, *cat*, *dapE*, *dat*, *ldh*, and *lhkA*) with at least one other genotype in the group (22). Clonal complexes were identified according to the predominant MLST type in the group. *In silico* MLST results were compared to results from SRST2 on short-read sequences (7) as described above, and conventional MLST results where known.

*In silico* typing was also performed on the reference genomes retrieved from GenBank and was compared to reported typing results (Table 1; see also Table S3 in the supplemental material).

**TABLE 1:** Concordance between predicted typing from WGS and conventional typing results

Surveillance type	*In silico* serotyping, no. (%)	*In silico* binary typing, no. (%)	*In silico* MLST, no. (%)
Retrospective (*n* = 423)	340/349 (97)^*a*^	319/346 (92)^*b*^	383/383 (100)
Prospective (*n* = 97)	92/96 (96)^*c*^	95/97 (98)^*d*^	97/97 (100)
Adjusted^*e*^ total (*n* = 520)	439/445 (99)	429/443 (97)	480/480 (100)
Reference genomes (*n* = 59)	53/55 (96)		22/22 (100)

a Includes 4 isolates with probable error in conventional typing result.

b Includes 22 isolates with probable error in conventional typing result.

c Includes 3 isolates with probable error in conventional typing result.

d Includes 1 isolate with probable error in conventional typing result.

e Adjusted totals include concordant results plus initially discordant results where repeat typing was concordant with the *in silico* result (i.e., probable error in the initial conventional-typing result).

### Prospective genomics-based surveillance.

Pilot surveillance of L. monocytogenes using WGS was undertaken over a 12-month period, with monthly analyses comparing isolates collected in a 12-month rolling window. Each isolate underwent *in silico* typing and core genome SNP analysis. To identify potential nested outbreak clusters, prospective isolates of the same lineage were analyzed with lineage-specific references (see Table S3 in the supplemental material). Clustered isolates in the window period prompted a more detailed core genome SNP-based analysis with historic isolates from the same MLST/clonal complex using a closely related reference genome. If no reference genomes from the same MLST/clonal complex were available, a high-quality *de novo* assembled draft genome of a local isolate was used. In consultation with epidemiologists from the national surveillance program, a system was developed where isolates were classified as being likely related, possibly related, or likely unrelated.

### Epidemiologic investigation.

Human cases and food source outbreaks of listeriosis were investigated by jurisdictional health departments, which included obtaining information on risk factors and food consumption during the exposure period where possible. Isolates retrieved from human clinical samples and from food and environmental testing were referred from the jurisdictional laboratories to the Microbiological Diagnostic Unit Public Health Laboratory.

Cases that resulted from consumption of a common food source where isolates retrieved from the clinical and food samples shared the same typing results by PFGE, MLST, MLVA, and PCR serotyping were considered to be epidemiologically linked.

### Nucleotide sequence accession number.

Raw sequence data have been uploaded to the European Nucleotide Archive (ENA) under the study accession no. PRJEB11543.

## RESULTS

### Whole-genome sequencing illustrates the inferences of the population structure from conventional typing.

**FIG 1:**
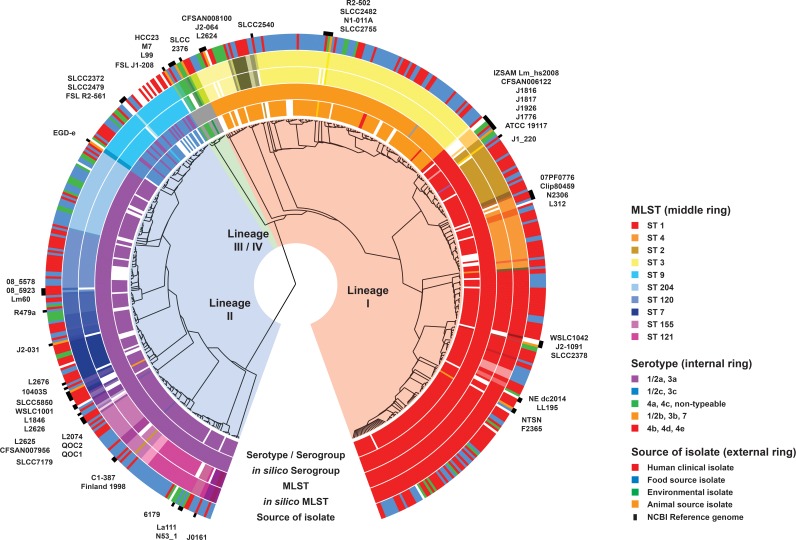
Maximum-likelihood phylogeny of Australian L. monocytogenes isolates. Isolates from human, food, and environmental sources are shown and include comparisons between three evolutionary lineages, serotypes, and MLSTs. The source of the isolate is indicated in the outer ring by the colors in the legend. The inner rings show serotype and MLST groupings, with the same color representing the same serotype or MLST in their respective rings. The most prominent MLST types are shown in the legend. Colors have been grouped to reflect lineage associations—lineage I (red, orange, yellow), lineage II (pink, purple, blue), and lineage III/IV (green). Similar colors (e.g., bright red and light red) also reflect closely related MLSTs that differ by a single allele, i.e., same clonal complex. The reference genomes listed in Table S3 in the supplemental material have been included for context. The phylogeny was inferred using FastTree v2.1.8 from the pairwise alignment of 158,707 core genome SNPs using the reference genome F2365 (BioSample accession no. SAMN02603980). The alignment was constructed using Snippy v2.5, and the figure was constructed using GraPhlAn v0.9.7 (https://huttenhower.sph.harvard.edu/graphlan).

A total of 520 L. monocytogenes isolates referred to the Microbiological Diagnostic Unit Public Health Laboratory from 1995 to 2015 were analyzed, including 423 retrospectively analyzed isolates and a further 97 isolates that were analyzed during a 12-month prospective surveillance period from 2014 to 2015. Raw sequencing metrics from WGS are shown in Table S1 in the supplemental material. Based on a phylogeny inferred from an alignment of 158,707 core genome SNPs, WGS revealed an Australian L. monocytogenes population structure derived from three distinct evolutionary lineages (Fig. 1).

The majority of Australian L. monocytogenes isolates were collected from either a food or a human source. Environmental isolates were included; however, they were usually collected as part of an outbreak investigation. A large proportion of the human isolates were serotypes 4b or 1/2a, although human, food, environmental, and animal isolates were found in every lineage. There were comparatively few isolates from lineage III, and there were no local Australian isolates from lineage IV.

Serotyping and MLST were reflective of the underlying phylogeny with clear WGS-based phylogenetic groups comprising MLST or serotype groups. For example, lineage I comprised serotypes 1/2b, 3b, 4b, 4d, 4e, and 7 while lineage II comprised serotypes 1/2a, 3a, 1/2c, and 3c. The phylogeny also supported clonal complex groupings with MLST profiles that differed by a single allele clustering together. However, the phylogeny also showed where isolates with different MLSTs were closely related, illustrating the limitations of categorical typing schemes, such as the traditional seven-gene MLST scheme (see Fig. S1 in the supplemental material).

### Analysis of the predominant clonal complex 1 illustrates the increased resolution of whole-genome sequencing over other typing methods.

**FIG 2:**
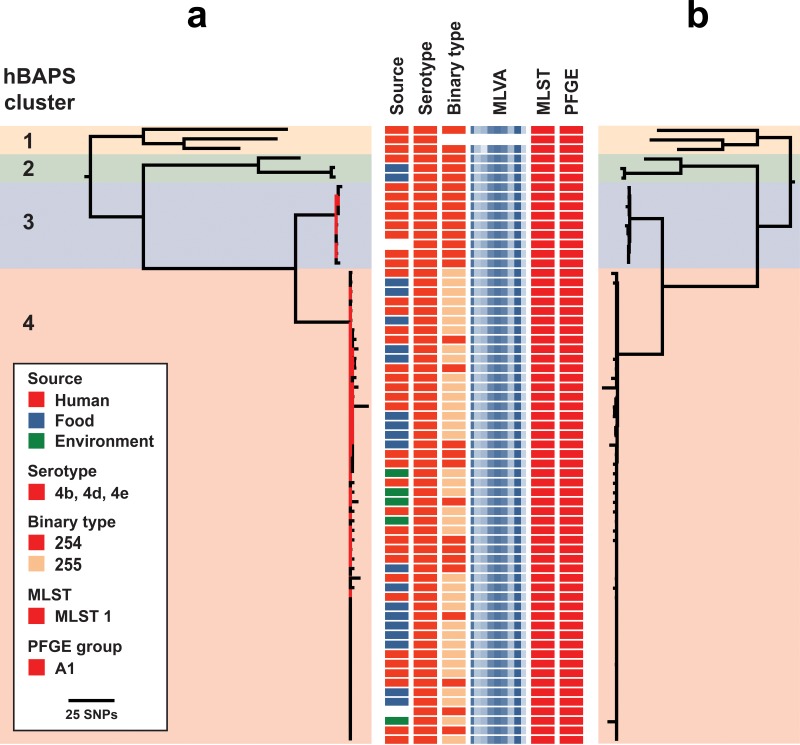
Core genome SNP phylogeny of isolates in PFGE group A1. Four distinct clusters are evident in (a) the maximum-likelihood phylogeny and (b) the neighbor-joining phylogeny, supported by hierarchical Bayesian estimations of the genetic population structure (hierBAPS). Isolates were indistinguishable by PFGE, MLST, and serotype. The maximum-likelihood tree was approximated using FastTree v2.1.8 with 1,000 bootstrap replicates. Major nodes had >99% bootstrap support, with nodes and branches with <70% support colored in red. SplitsTree4 was used to infer the neighbor-joining phylogeny from the alignment of core genome SNPs produced by Snippy v2.5. Core genome content was highly conserved (>90%) across these isolates.

One-quarter (*n* = 116) of the isolates were part of the clonal complex 1 (CC1) group, including sequence type 1 (ST1) isolates and ST1-like isolates differing by a single allele. Serotype, binary type, and MLVA patterns provided limited resolution to discriminate between isolates. Although several PFGE patterns were evident within the clonal complex 1 group, they also had less resolution to differentiate genetically similar isolates compared to those of WGS. For example, the core genome SNP-based phylogeny of one major group (PFGE pattern A1) contained four distinct clusters, supported by maximum-likelihood and hierarchical Bayesian estimations of the genetic population structure (Fig. 2). Cluster 4 represented an outbreak of L. monocytogenes involving food, environmental, and human clinical isolates. Although similar, no epidemiologic link was found between any of the isolates in cluster 3 and the isolates in the cluster 4 outbreak. These subclades included one distinct epidemiologically linked cluster of outbreak isolates that was otherwise indistinguishable from the other subclades with the conventional typing methods used.

**FIG 3:**
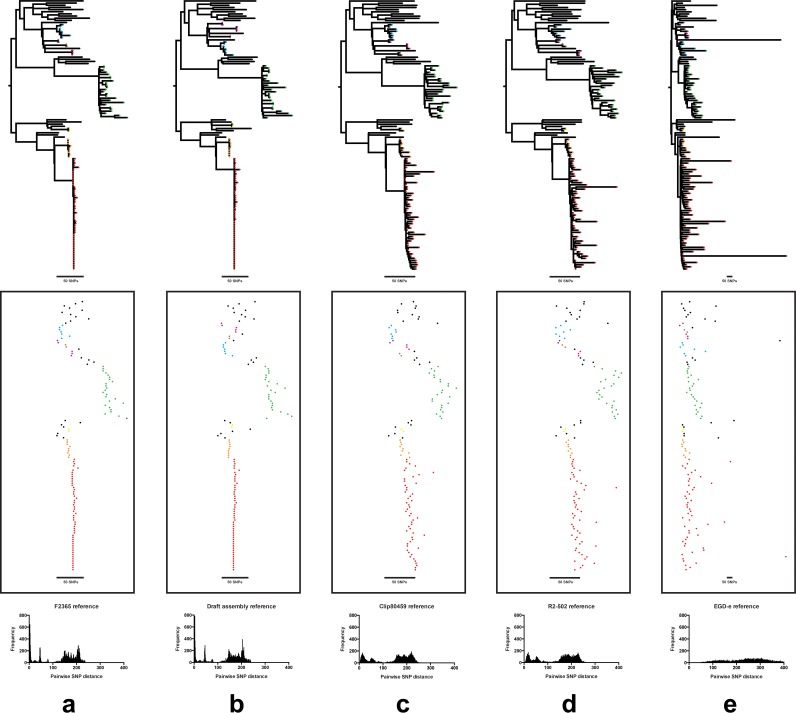
Comparison of the pairwise core genome SNP distances and resulting phylogeny for clonal complex 1 isolates when mapping to different reference genomes. The panels show the phylogeny (top), clusters of isolates (middle), and pairwise SNP distributions (bottom) when using (a) a closely related closed reference genome of the same MLST (F2365; BioSample accession no. SAMN02603980), (b) a local *de novo* assembled draft reference genome from the data set, (c) a closed reference genome of the same serotype, but different MLST (Clip 80459; BioSample accession no. SAMEA2272134), (d) a closed reference genome of the same lineage but different serotype and MLST (R2-502; BioSample accession no. SAMN02203126), and e) a closed reference genome from a different ancestral lineage (EGDe; Bio Sample accession no. SAMEA3138329). In the middle panel, the tips of the phylogenetic tree have been colored by cluster, with the branches obscured by the background.

Phylogenetic groupings based on tree structure were similar using rapid, but more rudimentary, neighbor-joining methods, compared to those based on more time-consuming maximum-likelihood approaches (Fig. 2). Similarly, compared to mapping against a closed CC1 genome, the use of a *de novo* assembled CC1 genome as a reference produced a similar reconstruction of the CC1 phylogeny and had sufficient resolution to facilitate detection of nested clusters (Fig. 3). In contrast, using the complete, but more genetically distant, EGD-e genome from lineage II for mapping lineage I CC1 isolates resulted in some loss of resolution due to the smaller number of shared (core genome) loci for SNP calling.

Although the same clusters of isolates were identifiable using different reference genomes, mapping to a closely related reference maximized the number of core genome sites available for SNP comparison and thus provided greater resolution to discriminate between two closely related isolates. Furthermore, some closely related isolates were falsely mapped against more distant references and incorrectly appeared unrelated due to the large number of false SNPs called (Fig. 3).

### WGS shows that outbreak isolates and isogenic isolates are not always identical.

**FIG 4:**
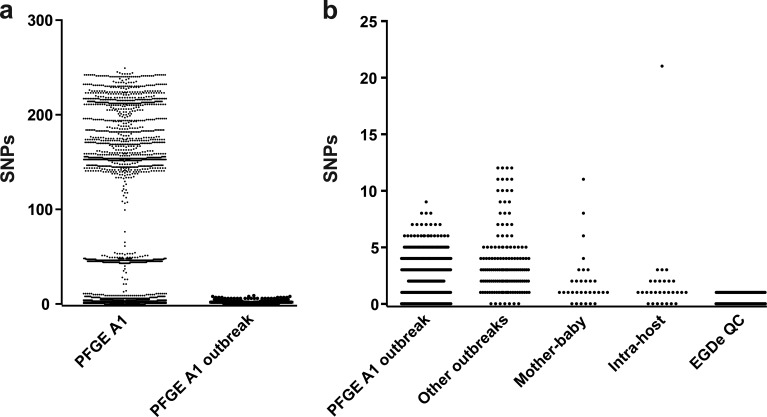
Pairwise SNP distances between closely related isolates. (a) Comparison of SNP distances between isolates in a single PFGE group (group A1) and between epidemiologically linked isolates within the same PFGE group. (b) Comparison of genetic diversity (measured in SNP distances between isolates) within epidemiologically linked outbreak groups, mother-baby paired isolates, isolates from a single host, and isolates from repeated sequencing of the reference strain EGD-e for quality control.

Within a single PFGE group (PFGE group A1), there was considerable genetic diversity with some isolates differing by over 200 SNPs (Fig. 4). However, isolates within this PFGE group that were epidemiologically linked differed by <10 SNPs. Analysis of other outbreak groups showed similar low diversity.

The majority of mother-baby paired isolates differed by <10 core SNPs, while multiple isolates from the same patient differed by <5 SNPs. One patient, whose isolates were collected a day apart and differed by 21 SNPs, was an exception. As a marker of quality control, the reference strain EGD-e (BioSample accession no. SAMEA3138329) was included in each sequencing run from 2012 to 2015. The strain was maintained through frozen storage with DNA re-extracted from a thawed sample every 3 months and with a single SNP emerging through this process.

### Routine WGS can be used for prospective surveillance of L. monocytogenes.

**FIG 5:**
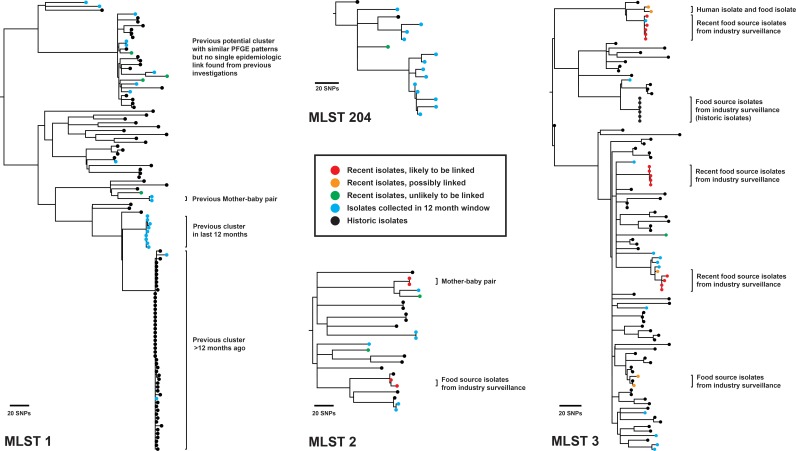
Prospective surveillance analysis of recent ST1, ST2, ST3, and ST204 isolates. Recent isolates collected in the last 1 month are compared with isolates collected in the preceding 12 months, with the analysis repeated every month. Historic isolates collected >12 months ago are also included for context. After comparison, recent isolates are labeled as likely to be linked, possibly linked, or unlikely to be linked. The results are interpreted together with epidemiologic information to refine relationships and potential links. Maximum-likelihood phylogenies based on core-genome SNPs are shown for MLST 1 (using the reference genome F2365; BioSample accession no. SAMN02603980), MLST 2 (reference genome J1-220; BioSample accession no. SAMN01813900), MLST 3 (reference genome R2-502; BioSample accession no. SAMN02203126), and MLST 204 (draft genome assembly of local ST204 isolate).

Phylogenetic analysis of 97 additional isolates sequenced during prospective surveillance revealed four potential clusters of isolates (MLST 1, 2, 3, and 204) for further investigation (Fig. 5). A single recent ST204 isolate was genetically distant from a small number of other ST204 isolates isolated in the preceding 12 months and was not thought to be epidemiologically linked (Fig. 5, green). This isolate also had a different PFGE pattern (PFGE C6) from the other ST204 isolates in the analysis (PFGE groups C1, C3, C4, AE1, AE2). Within the ST1 and ST2 groups, although a number of isolates were indistinguishable or closely related by PFGE, WGS analyses were not suggestive of a point source outbreak and subsequent independent epidemiologic investigations found no evidence to support an outbreak. However, isolates that were epidemiologically linked (e.g., a mother-baby pair) were revealed through WGS analysis. Analysis of ST3 isolates revealed 5 likely or potential clusters of linked isolates. Of these, 4 clusters were known to be from food industry sampling. A single human case was genetically similar to a food isolate and was epidemiologically investigated.

### Conventional typing results can be predicted using *in silico* tools.

One concern is the potential loss of backwards compatibility associated with a switch to WGS-based typing of pathogens relevant to public health. However, compared with those of conventional typing, *in silico* predictions of serotype, binary type, and MLST from WGS data were highly concordant (Fig. 1 and Table 1). For 6 of 13 (46%) isolates with discordant serotype results, there was clear evidence for the *in silico* result, which was supported by BLAST, read mapping, and phylogenetic inference, and the discordance was considered to be due to other errors, e.g., with conventional typing PCR sample mix-up. The results of repeat PCR serotyping of these six isolates were concordant with the *in silico* predictions and identified errors in the initial PCR typing results. Similarly, repeat binary typing for 15 of 29 (52%) discordant binary type results produced profiles consistent with those of the *in silico* predictions. When adjusted for these other errors, the concordance between conventional typing and *in silico* predicted results was 97% to 100%. The remaining discordant results in binary typing were due to insufficient read coverage across the locus (*n* = 4) and (>3) mutations in the forward primer (*n* = 2).

For serotyping, one discordant result was due to a break in the *de novo* assembly, and another was due to poor read coverage. The remaining 5 discrepancies were with resolving a particular phylogenetic clade in lineage II as serotypes 1/2a and 3a or 1/2c and 3c (Fig. 1). This was also evident from the reference strain EGD-e, reported to be serotype 1/2a but predicted *in silico* to be 1/2c or 3c. PCR serotyping also typed this strain as 1/2c or 3c. Almost all isolates in this clade were MLST 9 or clonal complex 9.

Of the reference genomes with known typing (see Table S3 in the supplemental material), *in silico* prediction of MLST attained 100% concordance with reported results, while *in silico* serotyping was concordant for 52/55 reference strains. Other than EGD-e, the discordant results were L1846, listed as serotype 1/2b on the NCBI GenBank site (BioSample accession no. SAMN02712416) but predicted to be 1/2a or 3a from *in silico* and phylogenetic analyses, and J2-1091, serotyped as 1/2a (BioSample accession no. SAMN02203123) but predicted to be 4b, 4d, or 4e.

## DISCUSSION

A number of proof-of-concept studies have alluded to the advantages of whole-genome sequencing (WGS) in public health microbiology for typing and outbreak investigation through clonality testing (5, 6, 23–28). However, of the few studies performed prospectively, the majority have been small in scale and have been performed over a short period of time to specifically investigate a putative outbreak, where strains are likely to be clonal. Our study reports the use of and provides a practical approach to routine WGS for epidemiologic surveillance in a national public health laboratory with a large data set that to our knowledge is the largest to report such comparisons with traditional typing of L. monocytogenes.

As the aforementioned studies have alluded to in the context of other bacterial pathogens, we found that WGS offered increased resolution to our existing typing methods for comparison of isolates, which allowed greater discrimination to infer the likelihood of transmission or a point source exposure in an outbreak (29–31). In addition, as our PFGE method was not identical to that described in the international PulseNet protocol, WGS provided a means to facilitate interlaboratory comparison with isolates from our laboratory using a high-resolution typing analysis (32). Furthermore, in our laboratory, which uses automated DNA extraction and library preparation, performing WGS was less expensive and less labor intensive per isolate than existing typing with PFGE, MLVA, MLST, and serotyping (data not shown).

Other studies have also reported the use of WGS to generate *in silico* typing data for organisms relevant to public health (7, 27, 33–36), but none have previously evaluated *in silico* predictions with results from conventional methods for a large data set of L. monocytogenes. The ability to accurately infer traditional typing information for L. monocytogenes and other pathogens of public health importance from WGS data can provide useful information for epidemiologists for retrospective comparisons in the transition from traditional to WGS-based characterization (27, 33). For this study, we developed bioinformatic tools to rapidly perform this retrospective comparison with 100% concordance for *in silico* MLST and 99% concordance for *in silico* PCR-serotyping compared with the wet-lab techniques currently used in our laboratory.

For reference-based methods, the choice of reference genome can significantly influence subsequent analyses (37, 38), and the lack of high-quality complete genomes for each phylogenetic group to serve as reference genomes may have been previously perceived to be a potential issue for accurate reference-based phylogenetic analysis and outbreak investigation. However, we found that the phylogeny inferred using a *de novo* assembled genome from within the group as a reference was similar to the phylogeny using a closely related completed genome. Therefore, this provides a viable alternative to using a distantly related reference genome. Notably, there are now more than 50 closed reference genomes in GenBank, including representative genomes from each serotype (see Table S3 in the supplemental material), and with rapid advances in technology, sequencing a pathogen genome to completion will soon become routine practice.

Comparisons of genetically distant groups of isolates by use of a single reference-based mapping method may lose some resolution, particularly if the selected reference is genetically distant to the isolates under investigation. To address this, after performing an initial analysis, we analyzed each cluster of closely related isolates, e.g., a single sequence type or clonal complex, separately using a closely related reference genome to maximize the accuracy and resolution of SNP calling. In comparing isolates across a long period of time, we included all relevant isolates in a new analysis, irrespective of whether they had been analyzed previously, to ensure the same comparator group of isolates and references.

Other nonmapping methods, such as those based on *de novo* assembly and annotation, have also been proposed and used for genomic comparisons of L. monocytogenes (39, 40). Although we did not use this in our analyses, a proposed core genome MLST (cgMLST) scheme based on a set of approximately 1,700 genes would provide an alternative high-resolution typing scheme to aid epidemiologic investigations. However, at the time of our study, cgMLST schemes for L. monocytogenes were only available commercially. In addition, some experts have argued that *de novo* assembly-based methods may have less resolution for SNP detection and can give misleading results particularly in repeat regions (N. Loman, presented at the 25th European Congress of Clinical Microbiology and Infectious Diseases, Copenhagen, Denmark, 25 to 28 April 2015) (41). We also speculate that mutations are less likely to emerge in core genome genes and therefore may not provide the same resolution as reference-based mapping, although large-scale comparison studies applying these methods in real-world situations are yet to be performed.

Other potential issues with using WGS have been raised. The current short-sequence read length prohibits the analysis of long repeat regions, which have previously been informative as a typing utility (e.g., MLVA) (42). However, we have demonstrated that effective analysis of phylogenetic relationships can be undertaken with short-read data, providing greater resolution for isolate discrimination than that of MLVA. Standards for the quality control of sequence data and bioinformatic analyses have yet to be defined and may be difficult to establish. In Australia, the Public Health Laboratory Network has proposed some recommendations for establishing WGS in public health microbial surveillance, but these are yet to be incorporated in national proficiency testing and accreditation programs (43).

Routine surveillance offers advantages over *ad hoc* WGS for suspected outbreaks. First, as with our study, it provides an overall sense of the genetic diversity of local strains, minimizing the selection bias that may accompany WGS employed specifically for an outbreak. In turn, this enhances knowledge of the local epidemiology of an organism, including prominent circulating local strains associated with human clinical disease. Furthermore, the inclusion of historic strains epidemiologically known to be linked or nonlinked to a current outbreak can be informative in determining the likelihood of a newly sequenced isolate being linked to the outbreak. Although some groups have attempted to define precise and absolute SNP thresholds for defining outbreak groups (31, 44), as others have also reported, in this study we found that these vary depending on a number of factors, including organism, reference genome selected, SNP calling parameters, and sequencing metrics (37, 38, 41). The number of SNPs may also be significantly influenced by the nature of the outbreak, with some being monoclonal point source outbreaks from a short period of time and others being prolonged or polyclonal outbreaks from a single source (30, 45–47), though we would argue that a polyclonal outbreak actually comprises a number of smaller outbreaks from a single source. As demonstrated in our analysis of clearly related strains, such as epidemiologically defined groups or mother-baby pairs, a range of SNP differences can be detected. We prefer to rely on phylogenetic comparisons with historic strains known to be involved or uninvolved in the current outbreak, where genomic clusters that include suspected outbreak and uninvolved historic strains are more likely to represent the emergence of a prominent clone rather than pathogen transmission or point source exposure.

The switch from the discrete categorical indexing of conventional typing methods to the more continuous and often less well-defined WGS methods to determine the relatedness of two isolates requires a different epidemiologic approach in analyzing the data. We found it difficult to define precise criteria for ruling in or ruling out isolates from an outbreak, and although we use an approximate guide specific to L. monocytogenes based on our experience (see Table S4 in the supplemental material), our definitions are used flexibly and cluster assignments are often subject to discussion. However, the reporting system for isolate relatedness that we used was found to be highly acceptable and interpretable by our epidemiologists, utilizing close communication between bioinformaticians, microbiologists, and epidemiologists to overlay traditional epidemiologic data upon genomic analyses to define outbreak clusters.

Finally, ongoing surveillance provides the opportunity to detect outbreaks in real time by monitoring the diversity of strains over time. Rapid detection of outbreaks is critical for limiting the spread of food-borne pathogens as well as agents of bioterrorism and multidrug-resistant organisms. After understanding how our retrospective WGS data would influence outbreak investigation, we successfully implemented a pilot methodology for prospective surveillance of L. monocytogenes in Australia. Having implemented this methodology, we have since moved to fortnightly analysis and reporting cycles, including all isolates from the potential incubation period for each cycle. Given our results, international collaborations such as the Global Microbial Identifier initiative (http://www.globalmicrobialidentifier.org/) and the COMPARE project (https://www.ebi.ac.uk/about/news/press-releases/compare-project-launch), together with platforms such as CLIMB (http://www.climb.ac.uk/) and GenomeTrakr (http://www.fda.gov/Food/FoodScienceResearch/WholeGenomeSequencingProgramWGS/ucm403550.htm) that provide frameworks for real-time global surveillance of organisms such as L. monocytogenes are well poised to be the future of public health microbial epidemiology.

## Supplementary Material

Supplemental material
