# Supplementary material for: Prospective Whole-Genome Sequencing Enhances National Surveillance of Listeria monocytogenes
Source: J Clin Microbiol. 2016 Jan 28;54(2):333–42. doi: 10.1128/JCM.02344-15 (PMC4733179; doi:10.1128/JCM.02344-15)
Supplement: Supplemental material [file supp_54_2_333__index.html]

Prospective Whole-Genome Sequencing Enhances National Surveillance of Listeria monocytogenes — Supplemental material 

# Prospective Whole-Genome Sequencing Enhances National Surveillance of Listeria monocytogenes

## Supplemental material

- Supplemental file 1 -

  Tables S1 (Raw sequencing metrics), S2 (Primer sequences for serogrouping), S3 (List of reference genomes used for comparison), and S4 (Guide criteria for assigning genomic relatedness) and Fig. S1 (Maximum likelihood phylogeny of clonal complex 1 isolates inferred from core genome SNPs)

  PDF, 643K
- Supplemental file 2 -

  Data Set S1 (Isolate information)

  XLSX, 223K
