# Supplementary material for: Prospective Whole-Genome Sequencing Enhances National Surveillance of Listeria monocytogenes
Source: J Clin Microbiol. 2016 Jan 28;54(2):333–42. doi: 10.1128/JCM.02344-15 (PMC4733179; doi:10.1128/JCM.02344-15)
Supplement: Supplemental material [file JCM.02344-15_zjm999094739so1.pdf]

SUPPLEMENTAL MATERIAL

Title: Prospective whole genome sequencing enhances national surveillance of *Listeria monocytogenes*

Authors: Jason C Kwong,[a,b,c] Karolina Mercoulia,[b] Takehiro Tomita,[b] Marion Easton,[b] Hua Y Li,[b] Dieter M Bulach,[a,d] Timothy P Stinear,[a,e] Torsten Seemann,[a,d] Benjamin P Howden[a,b,c,e] #

Affiliations: Doherty Centre for Applied Microbial Genomics[a] and Microbiological Diagnostic Unit Public Health Laboratory, University of Melbourne at the Doherty Institute for Infection & Immunity, Melbourne, Victoria, Australia,[b] Infectious Diseases Department, Austin Health, Heidelberg, Victoria, Australia,[c] Victorian Life Sciences Computation Initiative, University of Melbourne, Victoria, Australia,[d] Department of Microbiology, Monash University, Clayton, Victoria, Australia[e]

Keywords: *Listeria monocytogenes*, public health microbiology, whole genome sequencing, typing, pathogen surveillance

Running title: Prospective genomic surveillance of *L. monocytogenes*

TABLE S1: Raw sequencing metrics

	**Stored isolates**	**Prospective isolates**
**Read lengths (n)**	151 bp (n=80)<br>251 bp (n=212)<br>301 bp (n=131)	151 bp (n=7)<br>301 bp (n=90)
**Average per base read quality score [a]**	34.9 (31.1 – 37.2)	34.6 (33.2 – 36)
**Average read depth / coverage**	69.8 (30 – 206)	115.6 (71 – 314)

[a] Per base read quality scores:
10 = 1 error per 10 bases (90% accuracy);
20 = 1 error per 100 bases (99% accuracy);
30 = 1 error per 1000 bases (99.9% accuracy)

A minimum read depth of 30 and quality score >30 was considered acceptable.

TABLE S2: Primer sequences for Serogrouping

Gene name	Primer Name	Primer sequence (5´-3´)	PCR product size
lmo0737	LC3F	AGGGCTTCAAGGACTTACCC	691 bp
	LC3R	ACGATTTCTGCTTGCCATTC	
lmo1118	LC52F	AGGGGTCTTAAATCCTGGAA	906 bp
	LC52R	CGGCTTGTTCGGCATACTTA	
**ORF2110**	LB10F	AGTGGACAATTGATTGGTGAA	597 bp
	LB10R	CATCCATCCCTTACTTTGGAC	
**ORF2819**	LB50F	AGCAAAATGCCAAAACTCGT	471 bp
	LB50R	CATCACTAAAGCCTCCCATTG	
prs	LC32F	GCTGAAGAGATTGCGAAAGAAG	370 bp
	LC32R	CAAAGAAACCTTGGATTTGCGG	

**Serogroup**	***lmo1118*** **691 bp**	***lmo0737*** **906 bp**	**ORF2110 597 bp**	**ORF2819 471 bp**	***prs*** **370 bp**
**1/2a, 3a**	–	+	–	–	+
**1/2b, 3b, 7**	–	–	–	+	+
**1/2c, 3c**	+	+	–	–	+
**4b, 4d, 4e**	–	–	+	+	+
**Listeria spp.**					+

From Doumith et al, J Clin Microbiol 2004 (1)

TABLE S3: List of reference genomes used for comparison

ID	Assembly	BioProject	BioSample	Lineage	Serotype	MLST (CC)	Year isolated	Source	Metadata (Reference)
07PF0776	GCA_000258905.1	PRJNA78811	SAMN02604190	I	4b			Human	Myocardial abscess; human clinical, USA (2)
08_5578	GCA_000093125.2	PRJNA36361	SAMN02603776	II	1/2a		2008	Human	Canadian outbreak 2008; human blood, Canada (3)
08_5923	GCA_000022925.1	PRJNA36363	SAMN02603721	II	1/2a	120 (8)	2008	Human	Canadian outbreak 2008; human blood, Canada (3)
10403S	GCA_000168695.2	PRJNA17495	SAMN02641382	II	1/2a	85	1968	Human	Broad Institute, streptomycin resistant isolate of parental strain from 1968; human skin lesions, USA (4, 5)
6179	GCA_000577745.1	PRJEB1355	SAMEA3138937	II	1/2a	121		Environment	Cheese production environment; persistent strain (6, 7)
ATCC_19117	GCA_000307025.1	PRJEA51859	SAMEA2271997	I	4d	2		Animal	Sheep isolate, USA (8-10)
C1-387	GCA_000438605.1	PRJNA203445	SAMN02203127	II	1/2a		1999	Food	Isolated from turkey breast, New York, USA (11, 12)
CFSAN006122	GCA_000681515.1	PRJNA215355	SAMN02318985	I			2013	Food	FDA GenomeTrakr; cheese, USA (13)
CFSAN007956	GCA_001005985.1	PRJNA215355	SAMN02569973	II					FDA GenomeTrakr, USA (13)
CFSAN008100	GCA_001005925.1	PRJNA215355	SAMN02689388	I				Food	FDA GenomeTrakr; green chile ingredient, USA (13)
Clip80459	GCA_000026705.1	PRJEA32207	SAMEA2272134	I	4b	4	1999	Human	Rillettes epidemic; human clinical, France (14)
EGDe	GCA_000196035.1	PRJNA276	SAMEA3138329	II	1/2a	35	1924	Animal	Lab strain from rabbit isolate (15)
F2365	GCA_000008285.1	PRJNA85	SAMN02603980	I	4b	1	1985	Food	Jalisco cheese outbreak in California, USA (16)
FSL_J1-208	GCA_000250715.1	PRJNA53559	SAMN01121066	IV	4a			Animal	Outbreak in goat herds (17, 18)
FSL_R2_561	GCA_000168575.2	PRJNA17317	SAMN00013319	II	1/2c				(19)
Finland_1998	GCA_000168595.2	PRJNA17319	SAMN00012880	II	3a		1998	Human	Butter outbreak; food/clinical, Finland (20)
HCC23	GCA_000021185.1	PRJNA29409	SAMN02603154	III	4a			Animal	Avirulent strain from spleen of healthy channel catfish, USA (21, 22)
IZSAM_Lm_hs2008	GCA_000808055.1	PRJNA261392	SAMN03073546	I	4b		2009	Human	Cerebrospinal fluid (CSF), Italy (23)
J0161	GCA_000168635.2	PRJNA17493	SAMN00769971	II	1/2a		2000	Human	Human outbreak delicatessen turkey meat, USA (24)
J1776	GCA_000438705.2	PRJNA203445	SAMN02203125	I	4b		2002	Food	Turkey deli, USA (11)
J1816	GCA_000195395.3	PRJNA60535	SAMN01816124	I	4b		2002	Environment	Turkey deli, USA (11, 25)
J1817	GCA_000438725.2	PRJNA203445	SAMN02203129	I	4b		2002	Environment	Turkey deli, USA (11)
J1926	GCA_000438745.2	PRJNA203445	SAMN02203130	I	4b		2002	Human	Turkey deli, USA (11)
J1_220	GCA_000195435.3	PRJNA60537	SAMN01813900	I	4b		1979	Human	Outbreak with vegetables; human, Boston, USA (11, 25)
J2-031	GCA_000438645.1	PRJNA203445	SAMN02203124	II	1/2a		1996	Animal	Isolated from cow, USA (11)
J2-064	GCA_000438625.1	PRJNA203445	SAMN02203122	I	1/2b	5	1989	Animal	Isolated from cow, USA (11)
J2-1091	GCA_000438665.1	PRJNA203445	SAMN02203123	II	1/2a		1995	Animal	USA (12)

L1846	GCA_001027085.1	PRJNA243081	SAMN02712416	II	1/2b		2009	Human	Human clinical isolate; CDC, USA (26)
L2074	GCA_001027065.1	PRJNA243260	SAMN02713647	II	1/2a		2010	Human	Human clinical isolate; CDC, USA (26)
L2624	GCA_001027165.1	PRJNA243265	SAMN02713668	I	1/2b		2011	Human	Cantaloupe, human outbreak, USA (27)
L2625	GCA_001027125.1	PRJNA243264	SAMN02713648	II	1/2a		2011	Human	Cantaloupe, human outbreak, USA (27)
L2626	GCA_001027245.1	PRJNA243256	SAMN02713641	II	1/2a		2011	Human	Cantaloupe, human outbreak, USA (27)
L2676	GCA_001027205.1	PRJNA243258	SAMN02713644	II	1/2a		2011	Human	Cantaloupe, human outbreak, USA (27)
L312	GCA_000307085.1	PRJEA51857	SAMEA2272305	I	4b	4		Food	Cheese (9)
L99	GCA_000209755.1	PRJEA31327	SAMEA2272406	III	4a	201	1950	Food	Cheese, Netherlands (8, 9)
LL195	GCA_000318055.1	PRJEB418	SAMEA2272544	I	4b		1983-1987	Human	1983-1987 Switzerland outbreak; human clinical isolate, Switzerland (28)
La111	GCA_000382925.1	PRJEB538	SAMEA2272573	II	1/2a		1996	Food	Smoked salmon from fish processing plant, Denmark (29)
Lm60	GCA_000746625.1	PRJNA255073	SAMN03003922	II	1/2a	551	2006	Human	Isolated from blood, Switzerland (30)
M7	GCA_000218305.1	PRJNA66625	SAMN02604342	III	4a			Food	Non-pathogenic isolate from cow's milk, China (31)
N1-011A	GCA_000438685.2	PRJNA203445	SAMN02203128	I	1/2b			Environment	Environmental isolate, USA (32)
N2306	GCA_000950775.1	PRJNA276441	SAMN03372633	I	4b		2013-2014	Human	2013-2014 outbreak in ready-to-eat salads; human clinical isolate, Switzerland (33)
N53_1	GCA_000382945.1	PRJEB539	SAMEA2271966	II	1/2a		2002	Environment	Fish processing plant environment, Denmark (29)
NE_dc2014	GCA_000600015.1	PRJNA241322	SAMN02689320	I			2014	Food	USA (34)
NTSN	GCA_000800335.1	PRJNA267024	SAMN03177401	I	4b		2011	Animal	Sheep brain, China (35)
QOC1 *	GCA_000689335.1	PRJEB1353	SAMEA3139005	II	1/2a	403	2010	Human	Quargel outbreak clone 1, blood culture isolate, Austria (36)
QOC2 *	GCA_000613165.1	PRJEB1354	SAMEA3138995	II	1/2a	398	2010	Human	Quargel outbreak clone 2, blood culture isolate, Austria (36)
R2-502	GCA_000438585.1	PRJNA203445	SAMN02203126	I	1/2b		1994	Food	Illinois chocolate milk; food (11)
R479a	GCA_000613085.1	PRJEB5108	SAMEA3138991	II	1/2a	8	1996-1999	Food	Smoked salmon, Denmark (37)
SLCC2372	GCA_000210815.2	PRJEA50377	SAMEA2272627	II	1/2c	122 (9)	1935	Human	Human, UK (8, 9)
SLCC2376	GCA_000307065.1	PRJEA51863	SAMEA2272177	III	4c	71		Animal	Poultry (8, 9)
SLCC2378	GCA_000307615.1	PRJEA51729	SAMEA2272689	I	4e	73 (1)		Animal	Poultry (8, 9)
SLCC2479	GCA_000307005.1	PRJEA51829	SAMEA2272506	II	3c	9	1966		(8, 9)
SLCC2482	GCA_000210795.2	PRJEA50371	SAMEA2272162	I	7	3	1966	Human	Human (8, 9)
SLCC2540	GCA_000306905.1	PRJEA51731	SAMEA2272785	I	3b		1956	Human	Human, USA (8, 9)
SLCC2755	GCA_000197755.2	PRJEA50379	SAMEA2271969	I	1/2b	66 (3)	1967	Animal	Chinchilla (8, 9)

SLCC5850	GCA_000307045.1	PRJEA51861	SAMEA2272672	II	1/2a	12 (7)	1924	Animal	Rabbit, UK (8, 9)
SLCC7179	GCA_000306985.1	PRJEA51733	SAMEA2272571	II	3a	91	1986	Food	Cheese, Austria (8, 9)
WSLC1001	GCA_000568475.1	PRJNA236721	SAMN02743815	II	1/2a			Human	ATCC19112? (reportedly serotype 1/2c); human clinical isolate (CSF), Germany (38)
WSLC1042	GCA_000568935.1	PRJNA236791	SAMN02743817	I	4b				ATCC23074, Germany (38)

* Draft assembly – not used as a reference for analyses, but included for comparison

CC = Clonal Complex

Reference genomes retrieved from NCBI GenBank 22 June 2015.

Table S4: Guide criteria for assigning genomic relatedness (see additional notes below)

Relatedness	Criteria
**Likely to be linked**	1. pairwise SNP distance < 5 using a reference genome from the same MLST<br><br>OR (where there have been previous isolates epidemiologically linked to the index)<br><br>2. pairwise SNP distance less than the greatest pairwise SNP distance of a previous isolate epidemiologically linked to the index<br>AND<br>pairwise SNP distance less than that of each isolate not epidemiologically linked to the index
**Possibly linked**	1. pairwise SNP distance ≥ 5 to < 20 using a reference genome from the same MLST<br><br>OR (where there are previous isolates of the same MLST not epidemiologically linked to the index)<br><br>2. pairwise SNP distance ≥ 20<br>AND<br>more closely related to the index isolate than to previous "not epidemiologically linked" isolates
**Unlikely to be linked**	1. isolate from a different clonal complex to the index isolate<br><br>OR (where there have been previous isolates linked to the index)<br><br>2. pairwise SNP distance greater than that of another isolate previously known to be "not epidemiologically linked"<br>AND<br>pairwise SNP distance greater than that of each isolate linked to the index

Additional notes for Table S4:

- Isolates that cannot be assigned should be discussed, with the default assignment as "possibly linked" unless agreed otherwise
- The pairwise SNP distance threshold of < 5 for "likely to be linked" was selected, as the median SNP distance between isolates from previously defined outbreaks was 4, using the reported methods and parameters.
- The pairwise SNP distance threshold of 20 for "possibly linked" was selected, as this was the maximum SNP distance between isolates from previously defined outbreaks. This value is also consistent with previous reports (39).
- The criteria are intended as a guide for further epidemiologic analysis, and are not intended to be rigid. Exceptions can be made with sufficient evidence and agreement from epidemiologists, bioinformaticians and microbiologists.

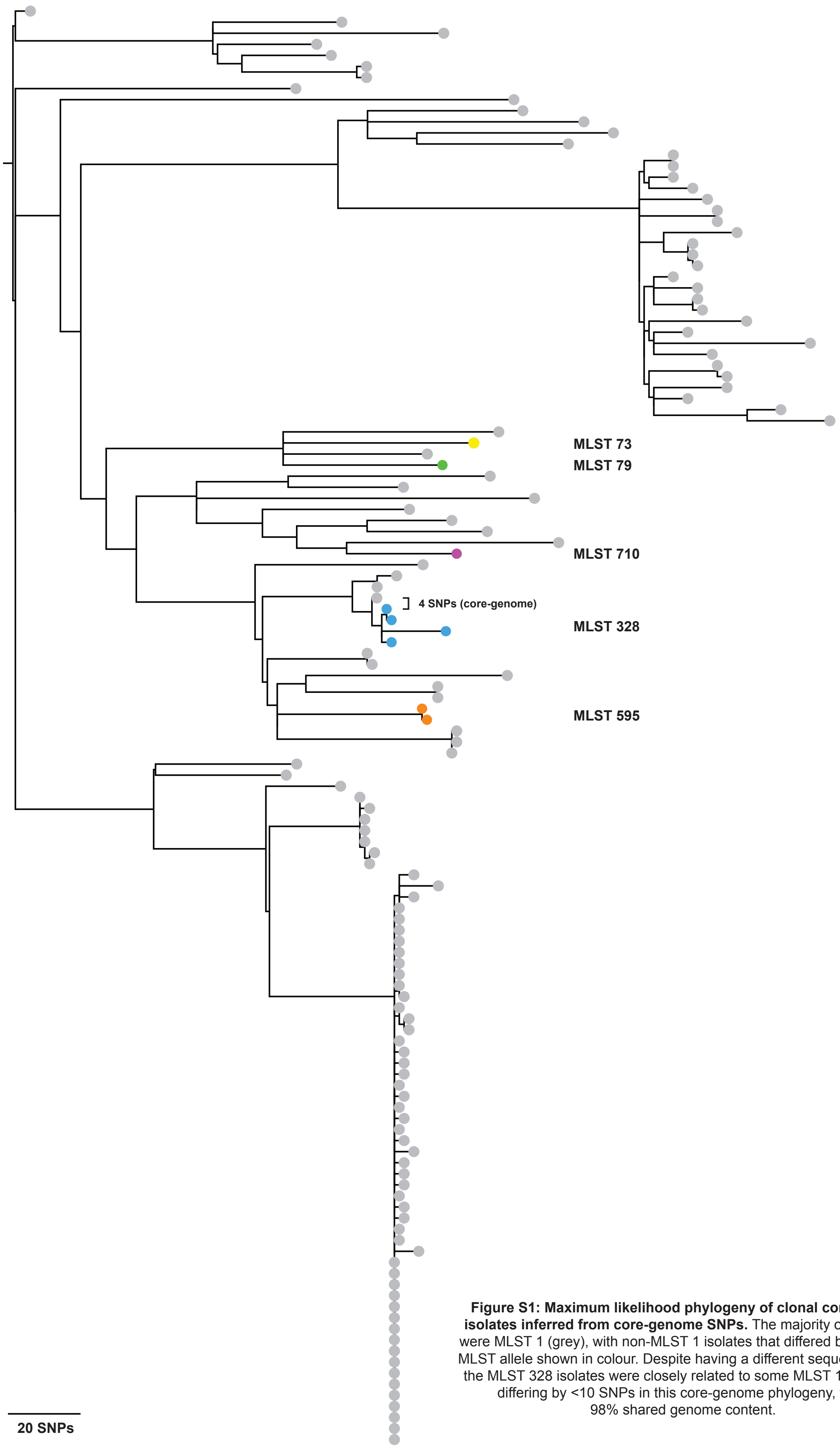

Figure S1: Maximum likelihood phylogeny of clonal complex 1 isolates inferred from core-genome SNPs. The majority of isolates were MLST 1 (grey), with non-MLST 1 isolates that differed by a single MLST allele shown in colour. Despite having a different sequence type, the MLST 328 isolates were closely related to some MLST 1 isolates, differing by <10 SNPs in this core-genome phylogeny, with 98% shared genome content.

REFERENCES

1. **Doumith M, Buchrieser C, Glaser P, Jacquet C, Martin P.** 2004. Differentiation of the major Listeria monocytogenes serovars by multiplex PCR. J Clin Microbiol **42:**3819-3822.
2. **McMullen PD, Gillaspy AF, Gipson J, Bobo LD, Skiest DJ, Freitag NE.** 2012. Genome sequence of Listeria monocytogenes 07PF0776, a cardiotropic serovar 4b strain. J Bacteriol **194:**3552.
3. **Gilmour MW, Graham M, Van Domselaar G, Tyler S, Kent H, Trout-Yakel KM, Larios O, Allen V, Lee B, Nadon C.** 2010. High-throughput genome sequencing of two Listeria monocytogenes clinical isolates during a large foodborne outbreak. BMC Genomics **11:**120.
4. **Bishop DK, Hinrichs DJ.** 1987. Adoptive transfer of immunity to Listeria monocytogenes. The influence of in vitro stimulation on lymphocyte subset requirements. J Immunol **139:**2005-2009.
5. **Edman DC, Pollock MB, Hall ER.** 1968. Listeria monocytogenes L forms. I. Induction maintenance, and biological characteristics. J Bacteriol **96:**352-357.
6. **Fox EM, Leonard N, Jordan K.** 2011. Physiological and transcriptional characterization of persistent and nonpersistent Listeria monocytogenes isolates. Appl Environ Microbiol **77:**6559-6569.
7. **Schmitz-Esser S RK, Mueller A, and Wagner M.** Institute for Milk Hygiene, University of Veterinary Medicine, Vienna, Austria. unpublished.
8. **Haase JK, Murphy RA, Choudhury KR, Achtman M.** 2011. Revival of Seeliger's historical 'Special Listeria Culture Collection'. Environ Microbiol **13:**3163-3171.
9. **Kuenne C, Billion A, Mraheil MA, Strittmatter A, Daniel R, Goesmann A, Barbuddhe S, Hain T, Chakraborty T.** 2013. Reassessment of the Listeria monocytogenes pan-genome reveals dynamic integration hotspots and mobile genetic elements as major components of the accessory genome. BMC Genomics **14:**47.
10. **Seeliger H.** 1961. Listeriosis. Hafner, New York,.
11. **Lomonaco S, Knabel SJ, Dalmasso A, Civera T, Bottero MT.** 2011. Novel multiplex single nucleotide polymorphism-based method for identifying epidemic clones of Listeria monocytogenes. Appl Environ Microbiol **77:**6290-6294.
12. **Roberson D CY, Xie Y, Thao K, Dao N, Clark TA, Boitano M, Korlach J, and Weimer B.** School of Veterinary Medicine, University of California at Davis, USA. unpublished.
13. **Timme R AM, Strain E, Evans PS, and Brown E.** Center for Food Safety and Applied Nutrition, US FDA, USA. unpublished.
14. **Hain T, Ghai R, Billion A, Kuenne CT, Steinweg C, Izar B, Mohamed W, Mraheil MA, Domann E, Schaffrath S, Karst U, Goesmann A, Oehm S, Puhler A, Merkl R, Vorwerk S, Glaser P, Garrido P, Rusniok C, Buchrieser C, Goebel W, Chakraborty T.** 2012. Comparative genomics and transcriptomics of lineages I, II, and III strains of Listeria monocytogenes. BMC Genomics **13:**144.
15. **Glaser P, Frangeul L, Buchrieser C, Rusniok C, Amend A, Baquero F, Berche P, Bloecker H, Brandt P, Chakraborty T, Charbit A, Chetouani F, Couve E, de Daruvar A, Dehoux P, Domann E, Dominguez-Bernal G, Duchaud E, Durant L, Dussurget O, Entian KD, Fsihi H, Garcia-del Portillo F, Garrido P, Gautier L, Goebel W, Gomez-Lopez N, Hain T, Hauf J, Jackson D, Jones LM, Kaerst U, Kreft J, Kuhn M, Kunst F, Kurapkat G, Madueno E, Maitournam A, Vicente JM, Ng E, Nedjari H, Nordsiek G, Novella S, de Pablos B, Perez-**

Diaz JC, Purcell R, Remmel B, Rose M, Schlueter T, Simoes N, et al. 2001. Comparative genomics of Listeria species. Science **294:**849-852.

16. **Nelson KE, Fouts DE, Mongodin EF, Ravel J, DeBoy RT, Kolonay JF, Rasko DA, Angiuoli SV, Gill SR, Paulsen IT, Peterson J, White O, Nelson WC, Nierman W, Beanan MJ, Brinkac LM, Daugherty SC, Dodson RJ, Durkin AS, Madupu R, Haft DH, Selengut J, Van Aken S, Khouri H, Fedorova N, Forberger H, Tran B, Kathariou S, Wonderling LD, Uhlich GA, Bayles DO, Luchansky JB, Fraser CM.** 2004. Whole genome comparisons of serotype 4b and 1/2a strains of the food-borne pathogen Listeria monocytogenes reveal new insights into the core genome components of this species. Nucleic Acids Res **32:**2386-2395.

17. **den Bakker HC, Bowen BM, Rodriguez-Rivera LD, Wiedmann M.** 2012. FSL J1-208, a virulent uncommon phylogenetic lineage IV Listeria monocytogenes strain with a small chromosome size and a putative virulence plasmid carrying internalin-like genes. Appl Environ Microbiol **78:**1876-1889.

18. **Wiedmann M, Mobini S, Cole JR, Jr., Watson CK, Jeffers GT, Boor KJ.** 1999. Molecular investigation of a listeriosis outbreak in goats caused by an unusual strain of Listeria monocytogenes. J Am Vet Med Assoc **215:**369-371, 340.

19. **Borowsky M YS, Zeng Q, Koehrsen M, Fitzgerald M, Alvarado L, Berlin A, Bochicchio J, Borenstein D, Chapman S, Chen Z, Engels R, Freedman E, Gellesch M, Goldberg J, Griggs A, Gujja S, Heilman E, Heiman D, Hepburn T, Towarth C, Jen D, Larson L, Lewis B, Mehta T, Park D, Pearson M, Roberts A, Saif S, Shead T, Shenoy N, Sisk P, Stolte C, Sykes S, Thomson T, Walk T, White J, Yandava C, Borodovsky M, Wiedmann M, Swaminathan B, Lauer P, Portnoy D, Cossart P, Buchrieser C, Higgins D, Haas B, Nusbaum C, and Birren B.** Broad Institute of MIT and Harvard, USA. unpublished.

20. **Lyytikainen O, Autio T, Maijala R, Ruutu P, Honkanen-Buzalski T, Miettinen M, Hatakka M, Mikkola J, Anttila VJ, Johansson T, Rantala L, Aalto T, Korkeala H, Siitonen A.** 2000. An outbreak of Listeria monocytogenes serotype 3a infections from butter in Finland. J Infect Dis **181:**1838-1841.

21. **Erdenlig S, Ainsworth AJ, Austin FW.** 2000. Pathogenicity and production of virulence factors by Listeria monocytogenes isolates from channel catfish. J Food Prot **63:**613-619.

22. **Steele CL, Donaldson JR, Paul D, Banes MM, Arick T, Bridges SM, Lawrence ML.** 2011. Genome sequence of lineage III Listeria monocytogenes strain HCC23. J Bacteriol **193:**3679-3680.

23. **Centorame P, Acciari VA, Orsini M, Torresi M, Iannetti L, Angius A, Di Giammartino D, Prencipe VA, Migliorati G.** 2015. Whole-Genome Sequence of Listeria monocytogenes Serovar 4b Strain IZSAM_Lm_hs2008, Isolated from a Human Infection in Italy. Genome Announc **3**.

24. **Olsen SJ, Patrick M, Hunter SB, Reddy V, Kornstein L, MacKenzie WR, Lane K, Bidol S, Stoltman GA, Frye DM, Lee I, Hurd S, Jones TF, LaPorte TN, Dewitt W, Graves L, Wiedmann M, Schoonmaker-Bopp DJ, Huang AJ, Vincent C, Bugenhagen A, Corby J, Carloni ER, Holcomb ME, Woron RF, Zansky SM, Dowdle G, Smith F, Ahrabi-Fard S, Ong AR, Tucker N, Hynes NA, Mead P.** 2005. Multistate outbreak of Listeria monocytogenes infection linked to delicatessen turkey meat. Clin Infect Dis **40:**962-967.

25. **Chen Y, Strain EA, Allard M, Brown EW.** 2011. Genome sequences of Listeria monocytogenes strains J1816 and J1-220, associated with human outbreaks. J Bacteriol **193:**3424-3425.

26. **Bergholz TM dBH, Turnsek M, Katz L, Joseph LA, Gladney LM, Kucerova Z, Silk B, Ward TJ, Frace M, Wiedmann M, and Tarr CL.** Centers for Disease Control and Prevention, USA. unpublished.

27. **Lomonaco S, Verghese B, Gerner-Smidt P, Tarr C, Gladney L, Joseph L, Katz L, Turnsek M, Frace M, Chen Y, Brown E, Meinersmann R, Berrang M, Knabel S.** 2013. Novel epidemic clones of Listeria monocytogenes, United States, 2011. Emerg Infect Dis **19:**147-150.

28. **Weinmaier T, Riesing M, Rattei T, Bille J, Arguedas-Villa C, Stephan R, Tasara T.** 2013. Complete Genome Sequence of Listeria monocytogenes LL195, a Serotype 4b Strain from the 1983-1987 Listeriosis Epidemic in Switzerland. Genome Announc **1**.

29. **Holch A, Webb K, Lukjancenko O, Ussery D, Rosenthal BM, Gram L.** 2013. Genome sequencing identifies two nearly unchanged strains of persistent Listeria monocytogenes isolated at two different fish processing plants sampled 6 years apart. Appl Environ Microbiol **79:**2944-2951.

30. **Tasara T, Weinmaier T, Klumpp J, Rattei T, Stephan R.** 2014. Complete Genome Sequence of Listeria monocytogenes Lm60, a Strain with an Enhanced Cold Adaptation Capacity. Genome Announc **2**.

31. **Chen J, Xia Y, Cheng C, Fang C, Shan Y, Jin G, Fang W.** 2011. Genome sequence of the nonpathogenic Listeria monocytogenes serovar 4a strain M7. J Bacteriol **193:**5019-5020.

32. **Chen Y, Zhang W, Knabel SJ.** 2007. Multi-virulence-locus sequence typing identifies single nucleotide polymorphisms which differentiate epidemic clones and outbreak strains of Listeria monocytogenes. J Clin Microbiol **45:**835-846.

33. **Tasara T, Ebner R, Klumpp J, Stephan R.** 2015. Complete Genome Sequence of Listeria monocytogenes N2306, a Strain Associated with the 2013-2014 Listeriosis Outbreak in Switzerland. Genome Announc **3**.

34. **Epie NN BM, Kan H-Y, Akanegbu C, and Diallo A.** Department of Forensic Sciences, DC Public Health Laboratory, USA. unpublished.

35. **Tan W, Wang G, Pan Z, Yin Y, Jiao X.** 2015. Complete Genome Sequence of Listeria monocytogenes NTSN, a Serovar 4b and Animal Source Strain. Genome Announc **3**.

36. **Rychli K, Muller A, Zaiser A, Schoder D, Allerberger F, Wagner M, Schmitz-Esser S.** 2014. Genome sequencing of Listeria monocytogenes "Quargel" listeriosis outbreak strains reveals two different strains with distinct in vitro virulence potential. PLoS One **9:**e89964.

37. **Schmitz-Esser S, Gram L, Wagner M.** 2015. Complete Genome Sequence of the Persistent Listeria monocytogenes Strain R479a. Genome Announc **3**.

38. **Klumpp J, Staubli T, Schmitter S, Hupfeld M, Fouts DE, Loessner MJ.** 2014. Genome Sequences of Three Frequently Used Listeria monocytogenes and Listeria ivanovii Strains. Genome Announc **2**.

39. **Wang Q, Holmes N, Martinez E, Howard P, Hill-Cawthorne G, Sintchenko V.** 2015. It Is Not All about Single Nucleotide Polymorphisms: Comparison of Mobile Genetic Elements and Deletions in Listeria monocytogenes Genomes Links Cases of Hospital-Acquired Listeriosis to the Environmental Source. J Clin Microbiol **53:**3492-3500.
